# A Rare Manifestation of Bullous Systemic Lupus Erythematosus in Children: A 10-year Retrospective Study in a Tertiary Care Hospital

**DOI:** 10.1155/2022/9388745

**Published:** 2022-07-19

**Authors:** Sunee Panombualert, Leelawadee Techasatian, Rattapon Uppala, Piti Ungareewittaya, Charoen Choonhakarn

**Affiliations:** ^1^Pediatric Department, Faculty of Medicine, Khon Kaen University, Khon Kaen, Thailand; ^2^Pathology Department, Faculty of Medicine, Khon Kaen University, Khon Kaen, Thailand; ^3^Medicine Department, Faculty of Medicine, Khon Kaen University, Khon Kaen, Thailand

## Abstract

Bullous systemic lupus erythematosus (BSLE) is an uncommon cutaneous presentation that occurs even less frequent in the pediatric population. A retrospective review was performed from January 2012 to December 2021 in all pediatric patients (aged <18 years) who fulfilled the diagnostic criteria for BSLE to evaluate the clinical characteristics, extracutaneous involvement, histopathologic features, immunofluorescence patterns, serological abnormalities, internal organ involvement, treatments, and outcomes. Among 1,415 patients with SLE, five patients were validated for the diagnosis of BSLE, accounting for 0.35%. The mean age at diagnosis was 12.2 years (standard deviation, 1.92). The clinical features of BSLE in the study population were generalized tense bullae and large extensive vesicles on the lips and perioral and mucosal areas. Pediatric BSLE in the study population revealed high SLE disease activity with multiple organ involvement. Hematologic abnormalities, serositis, and renal involvement were found in all patients, while polyarthritis (40%) and neurological abnormalities (40%) were less frequently observed. Systemic corticosteroids, intravenous immunoglobulin, immunosuppressants, antimalarials, and dapsone were prescribed in the study population. The cutaneous lesions subsided in all patients with a median clearance duration of 14 days (range, 5–56 days). BSLE in the pediatric population has auxiliary manifestations with high disease activity. Multiple organ involvement, especially hematologic abnormalities, serositis, and renal involvement, was frequently found in the study population. Although cutaneous lesions in BSLE subsided in all patients, involvement of other organs, especially renal impairment, required aggressive treatment, and long-term follow-up.

## 1. Introduction

Bullous systemic lupus erythematosus (BSLE) is a rare cutaneous condition. This manifestation mainly occurs in adult female individuals [1], and is even less frequent in the pediatric population [2]. Diagnosis of BSLE is challenging since similarities in the histology and immunopathology exist between BSLE and other primary bullous dermatoses, such as dermatitis herpetiformis (DH), linear IgA bullous dermatosis, and epidermolysis bullosa acquisita (EBA) [3–5]. However, clinical presentation combined with histology, immunological testing, and concomitant diagnosis of SLE distinguish this entity from other similar dermatoses [6]. Because of its rareness, the majority of previous publications were case reports. A previous study revealed a total of 10 BSLE patients during a 12-year retrospective review in the Thai adult population [7]; however, the literature on pediatric BSLE is scarce. The objective of this study was to perform a 10-year retrospective review of the pediatric population diagnosed with BSLE in the present tertiary care setting.

## 2. Methods

### 2.1. Study Design

The authors conducted a cross-sectional epidemiological study (10-year retrospective study) from January 1st, 2012, to December 31st, 2021, by collecting data from the medical records and the Health Object Program®, an authorized electronic medical records program, at the Srinagarind Hospital, Faculty of Medicine, Khon Kaen University, Thailand. All patients aged <18 years who fulfilled the diagnostic criteria for BSLE, and who visited the Srinagarind Hospital, Faculty of Medicine, Khon Kaen University, were included in the study. Collected data included clinical characteristics, extracutaneous involvement, histopathologic features, immunofluorescence patterns, serological abnormalities, internal organ involvement, treatments, and outcomes.

The diagnostic criteria for BSLE that were used for the diagnosis in the present study were the revised diagnostic criteria initial proposed by Camisa and Sharma (1983) [8], which included (1) a diagnosis of SLE based upon American Rheumatism Association criteria, (2) vesicles or bullae developing upon but not limited to sun-exposed skin, (3) histopathologic features of the lesional skin compatible with subepidermal blisters containing predominantly a neutrophil infiltration, similar to that of DH or inflammatory EBA, (4) negative or positive IIF for circulating BMZ antibodies using the split-skin technique, and (5) DIF of the lesional or nonlesional skin reveals linear or granular deposits of IgG and/or IgM and often IgA at the BMZ in case of the linear pattern deposition.

The study was approved by the Institutional Review Board of Khon Kaen University, Human Research Ethics Committee (#HE651011). The patients' informed consent forms and confirmation that all methods were performed in accordance with the relevant guidelines and regulations have been submitted to the journal.

### 2.2. Statistical Analysis

At the end of the study, the collected data were analysed using STATA software version 10 (StataCorp LP). Descriptive statistical methods, means, standard deviations (SDs), medians, and frequencies were used to analyse the demographic data.

## 3. Results

Among the 1,415 patients aged <18 years diagnosed with SLE, six developed vesiculobullous lesions. Five patients were validated for the diagnosis of BSLE, accounting for 0.35%. One patient with a vesiculobullous lesion was diagnosed with chronic bullous dermatosis of childhood (CBDC). Among the five BSLE patients, four were female (80%) and one was male. The mean age at diagnosis was 12.2 years old (SD, 1.92). Three of five developed BSLE simultaneously with the diagnosis of SLE. Two patients developed BSLE after SLE onset, with a mean onset of 35 days (10 days and 2 months). All patients fulfilled the criteria for the diagnosis of SLE using the European Alliance of Associations for Rheumatology (EULAR) and American College of Rheumatology (ACR) classifications. Constitutional symptoms with significant weight reduction before diagnosis were observed in all patients. Hematologic abnormalities, serositis, and renal involvement were found in all patients. Polyarthritis (40%) and neurological abnormalities (40%) were less frequently observed. The clinical presentation and laboratory findings of patients with BSLE are described in Table 1.

**Figure 1:**
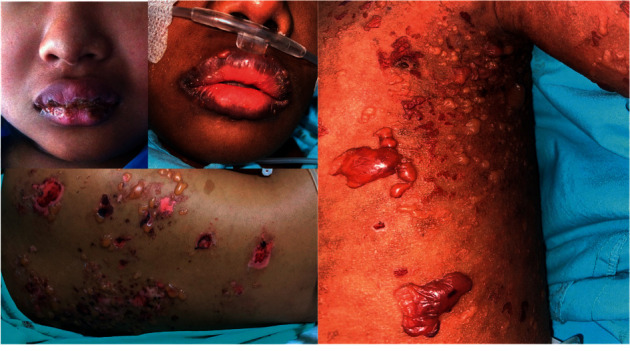
Generalized tense bullae, and extensive vesicles on the lips, perioral, and mucosal areas in patients with bullous systemic lupus erythematosus.

**Figure 2:**
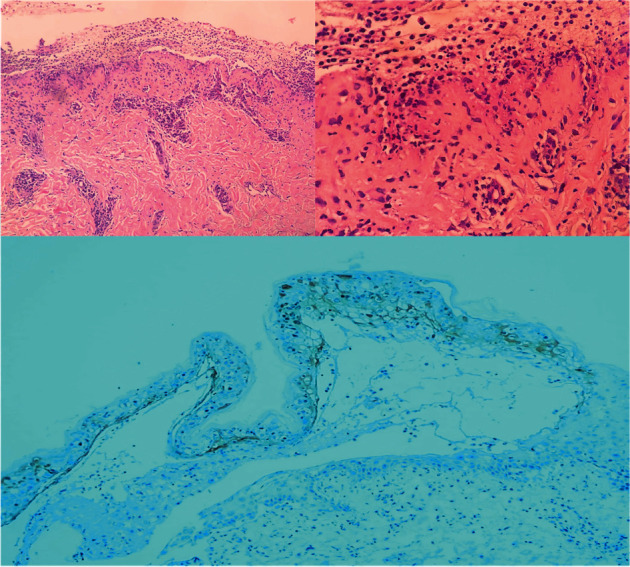
Subepidermal blister with neutrophils and interface dermatitis. Vacuolar alterations at the dermoepidermal interface with necrotic keratinocytes are presented (H&E ×40 and ×100). Immunohistochemical studies showed deposition of IgG at the dermoepidermal junction and within vessel walls.

The clinical manifestations of BSLE in the study population were generalized tense bullae, and some presented with large extensive vesicles on the lips, perioral, and mucosal areas (Figure 1).  Figure 2 shows the histopathology of cutaneous lesions of BSLE in the study population which revealed subepidermal blisters with neutrophils and interface dermatitis. Vacuolar alterations at the dermoepidermal interface with necrotic keratinocytes were also noted. We used direct immunofluorescence (DIF) to diagnose BSLE, but due to a technical issue with recording DIF photos, the authors were unable to produce DIF photographs of the presenting cases (a limitation of retrospective study design).  Figure 2 shows immunohistochemical staining of a leftover specimen in one of the cases described. Immunohistochemical studies showed deposition of IgG at the dermoepidermal junction and within vessel walls and C3 deposits at the dermoepidermal junction, with negative results for IgA and IgM. The other histopathological findings are described in Table 1.

Systemic corticosteroids, intravenous immunoglobulin (IVIG), immunosuppressants; mycophenolate mofetil, antimalarials, and dapsone were prescribed to treat BSLE in the study population. The cutaneous lesions subsided in all patients, with a median clearance duration of 14 days (range, 5–56 days). Long-term cutaneous hypopigmentation and discolouration of the involved areas were documented. Renal impairment was the remaining long-term consequence of pediatric BSLE which require aggressive immunosuppressant therapy and regular long-term follow-up.

## 4. Discussion

BSLE is a rare cutaneous manifestation of SLE, especially in the pediatric population [9]. The present study performed a retrospective study in a pediatric population and revealed five cases of BSLE, accounting for 0.35% of pediatric SLE cases during the past 10 years. This number was relatively high compared to the previous studies which were presented in case reports [1, 10–12]. This may be explained by the fact that the present study was performed in a referral center, dealing with severe and complicated pediatric SLE; thus, patients with uncommon or severe clinical presentations were transferred to this tertiary care center.

BSLE rarely presents as an initial or isolated manifestation [9, 13, 14]. This finding correlated with our setting, which revealed that none of the nondiagnosed SLE patients presented with isolated bullous lesions. Three of five (60%) patients exhibited BSLE simultaneously with the diagnosis of SLE and two of five (40%) developed BSLE later. The presentation of vesiculobullous lesions was found concurrently, or shortly after the diagnosis of SLE guided physicians to the final diagnosis of this rare condition [15]. However, bullous lesions in SLE should be distinguished from other bullous dermatoses [5], such as DH, EBA, CBDC, and leukocytoclastic vasculitis [16]. Therefore, BSLE should be confirmed by histology and immunopathology.

Some previous reports revealed that BSLE can be found even if the patient does not exhibit high SLE disease activity [14, 17]. This finding is in contrast to the present study which revealed high lupus disease activities with low complement levels in all patients (Table 1). The present study also revealed that multiple organ involvement was frequently found in patients with BSLE, especially hematologic abnormalities, serositis, and renal involvement, which were found in all of the study population. Serositis, including pleural and pericardial effusion, was extremely severe in this study population. Patients with renal involvement had lupus nephritis with active glomerulonephritis and required intensive and long-term immunosuppressants.

While systemic corticosteroids and immunosuppressants are the mainstay treatments for SLE, oral dapsone is frequently used in BSLE and it resolves cutaneous lesions [6, 12]. Several treatment modalities were prescribed in the study population due to the exhibition of high disease activity with severe multiple organ involvement. These include systemic corticosteroids, IVIG, immunosuppressants; mycophenolate mofetil, antimalarials, and dapsone. In recalcitrant cases of BSLE, rituximab was recently found to be an efficacious choice for patients who do not respond to dapsone or other immunosuppressants [6].

In a 12-year retrospective review, the majority of patients healed without scarring or milia, although postinflammatory hypo- or hyperpigmentation may be observed [7]. This finding is similar to the outcome of the present study which revealed complete clearance of the cutaneous lesion with a median duration of 14 days without BSLE recurrence. However, the remaining long-term consequence of pediatric BSLE in the study population was renal involvement which required aggressive immunosuppressant therapy and regular long-term follow-up.

## 5. Conclusions

In the present study, BSLE in the pediatric population had auxiliary manifestations that exhibited high disease activity. Multiple organ involvement, especially hematologic abnormalities, serositis, and renal involvement, were frequently found in the pediatric population. Although cutaneous lesions in BSLE subsided in all patients, the remaining long-term consequence of pediatric BSLE was renal involvement which required aggressive immunosuppressant therapy and regular long-term follow-up.

### 5.1. Limitation

The main limitation of the present study was its retrospective design, which resulted in a lack of certain information, such as pathological and immunofluorescent pictures. However, all available documents fulfilled the diagnostic criteria for BSLE, a rare presentation in the pediatric population.

## Figures and Tables

**Table 1:** Description of clinical characteristic, laboratory findings, treatments, and outcomes of pediatric BSLE patients in the study population.

Category	No. 1	No. 2	No. 3	No. 4	No. 5
Age at diagnosis (years)	10	11	13	12	15
Gender	F	M	F	F	F
Constitutional symptoms/weight loss	Yes	Yes	Yes	Yes	Yes

Cutaneous lesion					
(i) Vesiculobullous pattern	Generalized tense bullae with varying sizes on the face, trunk, back, and extremities	Generalized tense bullae with varying sizes on the face, lips, trunk, back, and genitalia	Multiple tense bullae on the lips, perioral, and genital area	Multiple tense bullae on the lips, mucosa, and genital area	Large and multiple tense bullae on the lips and perioral area
(ii) Oral ulcer	Yes	Yes	Yes	Yes	Yes
(iii) Mucosal lesions	Yes	Yes	Yes	Yes	Yes
(iv) Other cutaneous manifestation	No	Cutaneous vasculitis, palmar erythema	Malar rash	Discoid rash on the scalp with scarring alopecia	No

Histopathological tissue compatible with SLE					
(i) Skin	Yes	Yes	No	No	Yes
(ii) Kidney	No	No	Yes	Yes	No
(iii) Other tissue	No	No	No	Pericardium	No

Systemic involvement					
Neurological involvement	No	Yes (Seizure)	Yes (Seizure)	No	No
Polyarthritis	No	No	No	Yes	Yes
Serositis	Yes (pleural effusion)	Yes (pleural effusion)	Yes (pleural and pericardial effusion)	Yes (pericardial effusion with cardiac tamponade)	Yes (pleural effusion)
Hematological abnormalities	Yes	Yes	Yes	Yes	Yes
(i) AIHA	Yes	Yes	Yes	Yes	Yes
(ii) Lymphopenia	Yes	Yes	Yes	Yes	Yes
(iii) Thrombocytopenia	No	Yes	Yes	Yes	Yes
(iv) MAS/HLH	No	Yes	No	No	No
(v) DCT	Negative	Positive	Positive	Positive	Positive
Renal involvement	Yes	Yes	Yes (LN class IV)	Yes (LN class IV)	Yes
(i) Hypertension	Yes	Yes	Yes	Yes	Yes
(ii) BUN/Creatinine	72.7/2.73	5.3/1.73	33/1.4	26.9/1.77	23/1.07
(iii) Proteinuria	Yes (protein 4+)	Yes (protein 1+)	Yes (protein 3+)	Yes (protein 2+)	Yes (protein 3+)
(iv) Glomerulonephritis	Yes (RBC 10–20)	Yes (RBC 5–10)	Yes (RBC 20–30)	Yes (RBC 30–50)	Yes (RBC 10–20)
(v) UPCR (mg/mg)	3	5.7	7.5	3.7	2
(Normal range <0.2)					

Complement levels					
(i) C3 (normal range 90–180)	Low (52.9)	Low (9.8)	Low (12.3)	Low (22)	Low (18)
(ii) C4 (normal range 10–40)	Low (10.7)	Low (0.6)	Low (6)	Low (7)	Low (5)
Vitamin D level (normal range >20)	Low (8.24)	Low (23	Deficiency	N/A	N/A
Serum albumin (g/dl)	3.2	2.1	2.4	3.3	3.0

ANA	Positive	Positive	Positive	Positive	Positive
	Coarse speckle type	Coarse speckle type	Homogenous type	Homogenous type	Coarse speckle type
	1 : 2560	1 : 2560	1 : 1280	1 : 5120	1 : 2560

Anti-dsDNA	Positive	Positive	Positive	Positive	Positive
	720 IU/ml	104.8 IU/ml			

Anti-smith	Positive	Negative	N/A	N/A	N/A
	116 IU/ml				

Treatments	Dapsone	Dapsone	Prednisolone	Dapsone	Dapsone
	Prednisolone	IVIG	MMF	Prednisolone	Prednisolone
	MMF	Prednisolone	CP	MMF	HCQ
	HCQ	MMF	HCQ	HCQ	
		HCQ			

Outcomes					
(i) Cutaneous clearance (days)	14	56	7	5	10
(ii) Long-term cutaneous lesions	Discolouration hypopigmentation on the involved area	Discolouration on the involved area	Hypopigmentation on the involved area	Complete recovery	Complete recovery

(iii) Renal impairment	Yes	Yes	Yes	Yes	Yes
	LN	LN	LN	LN	LN

(iv) Hematological abnormalities	Improve	Improve	Recovery	Recovery	Recovery

AIHA, autoimmune induced hemolytic anemia; ANA, antinuclear antibody; Anti-dsDNA, anti-double stranded DNA; BUN, blood urea nitrogen; CP, cyclophosphamide; DCT, direct Coombs' test; HCQ, hydroxychloroquine; HLH, hemophagocytic lymphohistiocytosis; IVIG, intravenous immunoglobulin; LN, lupus nephritis; MAS, macrophage activation syndrome; MMF, mycophenolate mofetil; N/A, not available; UPCR, urine protein/creatinine ratio.

## Data Availability

The data and materials used and/or analysed during the current study are available from the corresponding author on reasonable request.

## Abbreviations:

*SLE*: Systemic lupus erythematosus
*BSLE*: Bullous systemic lupus erythematosus
*CBDC*: Chronic bullous dermatosis of childhood
*DH*: Dermatitis herpetiformis
*EBA*: Epidermolysis bullosa acquisita.
